# MBenes for Advanced Water Treatment and Desalination Membranes: A Bibliometric, Materials Engineering, and Future Perspectives Review

**DOI:** 10.3390/membranes16080258

**Published:** 2026-07-28

**Authors:** Asam Amin Almulla, Fikri T. Dweiri

**Affiliations:** Department of Industrial Engineering and Engineering Management, University of Sharjah, Sharjah 27272, United Arab Emirates

**Keywords:** MBenes, two-dimensional materials, desalination, water treatment, ultrafiltration membranes, bibliometric analysis

## Abstract

Water scarcity and rising demand for safe water continue to motivate the development of improved treatment and desalination membranes. MBenes are an emerging family of two-dimensional transition-metal borides derived from layered MAB phases. Material-level experiments and computational studies indicate potentially useful electronic, structural, and surface properties; however, direct evidence for MBene membranes remains limited. This review combines an author-generated bibliometric analysis of 279 Scopus-indexed records, retrieved on 14 April 2026, with a critical assessment of MBene synthesis, properties, and prospective membrane applications. Evidence from graphene, graphene oxide, transition-metal dichalcogenides, and MXenes is treated as analogous evidence rather than direct validation of MBene membrane performance. The analysis shows that MBene research is concentrated mainly in synthesis, computational modeling, catalysis, and energy storage, whereas experimentally validated water-treatment and desalination membranes are scarce. Priorities include reproducible MBene synthesis, controlled membrane fabrication, standardized performance testing, long-term stability and leaching assessment, and pilot-scale and techno-economic evaluation.

## 1. Background

Water scarcity affects a substantial proportion of the global population and is intensified by demographic growth, urbanization, industrial activity, and climate variability [1,2,3,4,5,6,7,8,9,10,11,12,13]. Desalination is therefore important in water-stressed regions that depend on seawater or brackish groundwater [14,15]. Reverse osmosis (RO) is the dominant modern desalination process because it is generally less energy-intensive than conventional thermal desalination and can be implemented across a range of plant capacities [1,2,14,15].

RO nevertheless remains constrained by energy demand, membrane fouling, chemical cleaning, membrane replacement, and the management of concentrated brine [16,17,18]. Global desalination also produces a brine stream larger than the volume of freshwater generated, emphasizing the need for responsible discharge and resource-recovery strategies [16,17,18]. In ultrafiltration (UF), natural organic matter can adsorb on membrane surfaces and within pores, causing irreversible permeability loss [16,17,18]. Hydrophilic surface modification and nanomaterial incorporation are, therefore, widely investigated to improve wettability and fouling resistance, although the outcome depends on membrane chemistry, morphology, feed composition, and operating conditions [16,17,18].

Two-dimensional materials, including graphene derivatives, transition-metal dichalcogenides (TMDs), and MXenes, have expanded the range of structures available for membrane design [19,20,21,22]. MXenes are two-dimensional transition-metal carbides, nitrides, or carbonitrides whose surface terminations and interlayer spacing can be modified [9,19,20,21,22]. Experimental Ti_3_C_2_T_x_ membrane studies have demonstrated ion sieving, aqueous transport, and antibacterial activity under specific configurations; these findings support MXenes as membrane benchmarks [19,20,21,22].

This review examines the prospective role of MBenes in water-treatment and desalination membranes [23,24,25,26,27]. Established membrane processes include microfiltration, UF, nanofiltration, and RO, and polymeric membranes such as polysulfone (PSU) and polyethersulfone (PES) are frequently modified to improve hydrophilicity, permeability, and fouling resistance [23,24,25,26,27]. Reported improvements are system-specific and cannot be transferred directly to MBene-polymer membranes without experimental validation [23,24,25,26,27].

Silver-nanoparticle-modified polymeric membranes provide a separate benchmark for antimicrobial membrane design [28,29,30]. Experimental PSU and thin-film composite membranes containing immobilized silver have shown reduced bacterial attachment or biofouling, but silver leaching, long-term activity, and environmental safety require careful evaluation [28,29,30]. These studies are included as general antifouling evidence and not as MBene evidence [28,29,30].

Because experimentally validated MBene membrane studies are scarce, this review uses four evidence categories: (i) direct experimental MBene evidence; (ii) computational MBene evidence; (iii) analogous evidence from MXenes, graphene, graphene oxide, TMDs, and other membrane materials; and (iv) prospective applications that remain unvalidated [31,32,33,34,35]. Analogous evidence is used to identify potentially transferable mechanisms and design principles, not as proof of equivalent MBene membrane performance [31,32,33,34,35].

## 2. Bibliometric Methodology and Global Research Landscape of MBenes (2017–2026)

The bibliometric results reported in this review were calculated by the authors from a Scopus dataset exported on 14 April 2026 using the advanced search query: TITLE-ABS-KEY (MBene OR “Two-Dimensional Transition Metal Boride” OR “Transition Metal Boride Nanosheet”). The search retrieved 279 documents published between 2017 and 2026. Following data cleaning and verification, all 279 retrieved records were retained for the descriptive bibliometric analysis. The annual publication counts, document types, subject-area distributions, keyword frequencies, and network results presented in this section were generated directly by the authors from the exported Scopus records and were not obtained from previously published reviews. The dataset was checked for duplicate records, incomplete bibliographic information, and consistency in keywords and material terminology before analysis. No records were excluded during this process.

### 2.1. Bibliometric Data Collection and Analysis Methodology

#### 2.1.1. Database, Search Strategy, and Data Retrieval

The bibliometric analysis was conducted using Scopus. The final search was performed on 14 April 2026 using the advanced query: TITLE-ABS-KEY (MBene OR “Two-Dimensional Transition Metal Boride” OR “Transition Metal Boride Nanosheet”). The search covered publications indexed in Scopus between 2017 and the search date, which retrieved 279 records. No restrictions were applied by country, institution, subject area, or document type. Because the search was conducted in April 2026, the publication count for 2026 represents a partial year and should not be interpreted as a complete annual output.

#### 2.1.2. Inclusion, Exclusion, and Screening Criteria

Records were eligible when their title, abstract, or keywords contained “MBene,” “two-dimensional transition metal boride,” or “transition metal boride nanosheet.” Research articles, review articles, book chapters, conference papers, errata, and other Scopus-indexed document types were considered because the analysis aimed to map the complete development of the emerging MBene research field. Records were excluded if they were duplicates, lacked sufficient bibliographic information for analysis, or used the search terms in a context unrelated to two-dimensional transition-metal borides. Screening was performed by examining titles, abstracts, author keywords, index keywords, document types, and available bibliographic information. The initial search identified 279 records. No duplicate or ineligible records were identified; therefore, all 279 records were retained for the descriptive bibliometric analysis.

#### 2.1.3. Data Cleaning and Descriptive Analysis

The Scopus records were exported with complete citation information, bibliographic information, abstracts, author keywords, index keywords, affiliations, references, and funding details where available. Duplicate records were checked first using DOI and then by comparing publication titles. Keyword cleaning involved standardizing capitalization, singular and plural forms, hyphenation, abbreviations, and closely related terms. Variants such as “MBene” and “MBenes” were merged, while MXenes and MBenes were retained as separate material categories. Terms with distinct technical meanings were not merged.

Annual publication counts, document types, subject areas, countries, institutions, and source titles were calculated directly by the authors from the cleaned Scopus dataset. Publication year was determined using the year recorded in Scopus. Subject-area counts were treated as non-exclusive because one publication may be assigned to more than one Scopus subject area; consequently, the sum of the subject-area counts may exceed the total number of 279 records.

#### 2.1.4. VOSviewer Network Analysis

Keyword co-occurrence analysis was conducted using VOSviewer version 1.6.20 [36]. The analysis type was co-occurrence, the unit of analysis was all keywords, and full counting was used. The minimum occurrence threshold was five, and every keyword meeting that threshold was included. No thesaurus file was applied. Association-strength normalization was used with a clustering resolution of 1.00 and a minimum cluster size of one [36]. The resulting network contained three clusters. Node size represents keyword occurrence, link thickness indicates total link strength, and node color identifies the assigned thematic cluster [36].

#### 2.1.5. Verification of Bibliometric and Technical Data

Two types of numerical evidence are reported in this review. First, the bibliometric counts were calculated by the authors directly from the Scopus dataset. These include the total number of documents, annual publication counts, document types, subject-area distributions, keyword occurrences, and VOSviewer network indicators. All bibliometric results were cross-checked against the cleaned Scopus dataset and the corresponding VOSviewer outputs. Second, quantitative membrane and material-performance values were extracted from published studies. Each retained technical value was checked against its cited source and classified according to the investigated material and evidence type. Values originating from MXenes, graphene, graphene oxide, transition-metal dichalcogenides, or general membrane studies were treated as benchmark or analogous evidence rather than direct MBene evidence. Numerical values that could not be traced to a specific source or appropriately linked to the investigated material were removed or replaced with qualified qualitative statements.

### 2.2. Publication Growth and Research Distribution

The retrieved records show low annual output between 2017 and 2020, followed by marked growth from 2021 onward. The recorded counts include 20 publications in 2021, 39 in 2023, 76 in 2024, and 110 in 2025. Only three records were indexed for 2026 by the retrieval date; however, because the search was performed on 14 April 2026, this value represents a partial year and should not be compared with complete annual totals. The literature associates the early period with theoretical prediction and initial synthesis, followed by broader investigation of electrochemical, catalytic, and environmental applications [3,4,5,6,7,8,12,37].

### 2.3. Subject-Area Distribution and Interdisciplinary Nature

Within the dataset, Materials Science accounted for 177 records, followed by Chemistry (137), Physics and Astronomy (95), Engineering (86), and Chemical Engineering (74). Scopus subject areas are non-exclusive, so their counts exceed the total number of documents. The distribution indicates that MBene research spans materials characterization, computational modeling, chemistry, and engineering applications.

### 2.4. Publication Outlets and Research Types

The dataset contained 251 research articles and 22 reviews, together with a small number of other Scopus-indexed document types. Frequently represented sources included *Advanced Functional Materials*, *Chemical Engineering Journal*, *Journal of Colloid and Interface Science*, *Small*, and *Nanoscale*. These descriptive results identify publication outlets and document types; they are not used as indicators of study quality or field maturity.

### 2.5. Emerging Trends and Challenges

**Figure 1:**
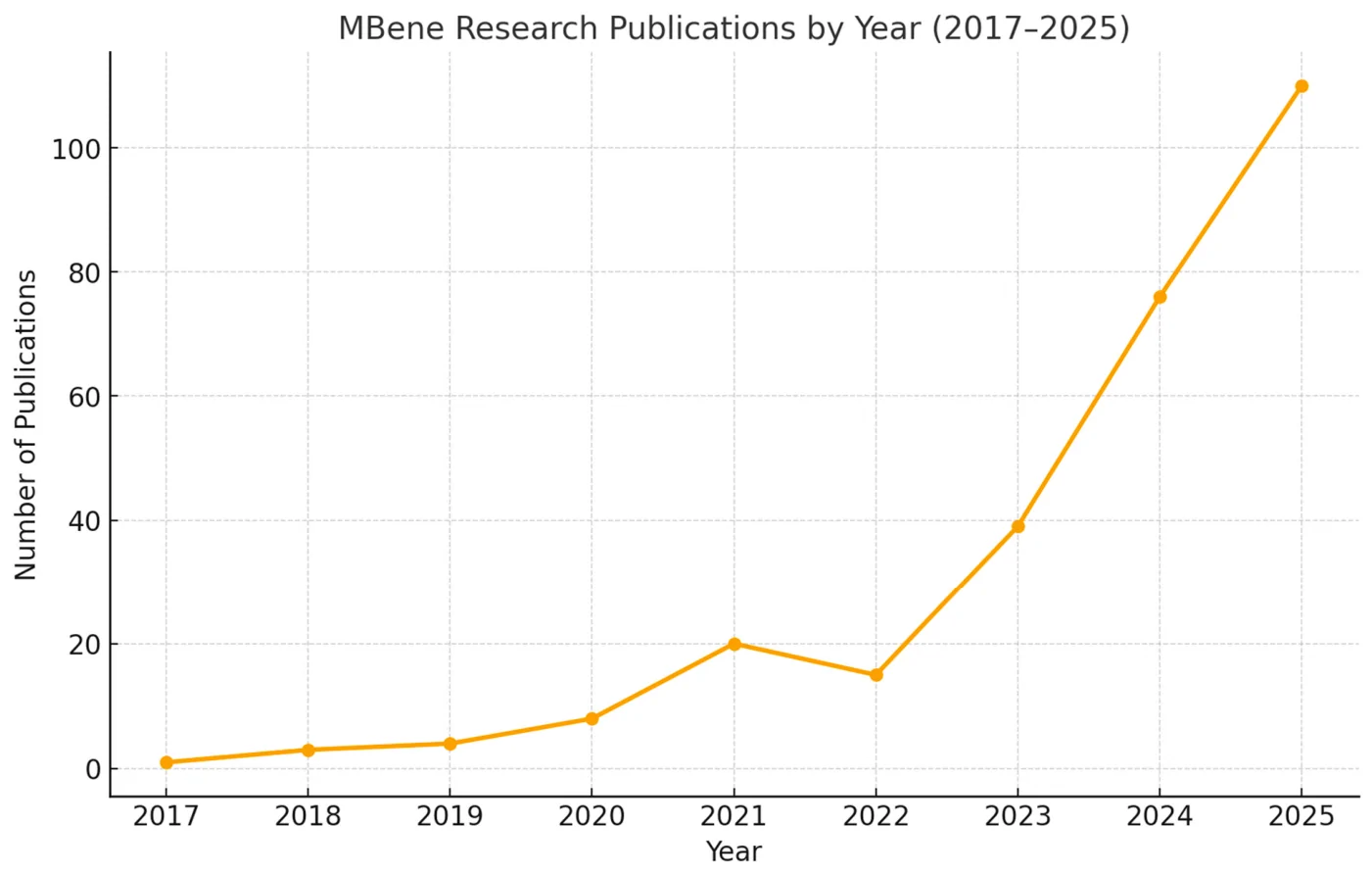
Annual MBene-related publications in 279 Scopus records retrieved on 14 April 2026; 2026 data are incomplete.

As shown in Figure 1, publication output increased substantially after 2020. Keyword and source-title patterns indicate continuing activity in DFT modeling alongside growth in experimental synthesis, electrochemistry, catalysis, and functionalization. These patterns describe the composition of the retrieved dataset and do not, by themselves, establish technological readiness.

However, the field still faces challenges [3]. The synthesis of phase-pure MBenes remains nontrivial due to the strength of M-B bonds and the difficulty of controlled A-layer removal [5,6,7]. Moreover, while computational predictions of performance are abundant, scalable experimental production and long-term stability testing are comparatively scarce [38,39]. Bridging this gap will likely define the next stage of research, pushing MBenes from theoretical feasibility to industrial relevance [38,39].

MBenes are two-dimensional transition-metal borides associated with layered MAB precursors [40,41]. Depending on composition and synthesis route, the A-layer may be selectively removed or a two-dimensional boride may be obtained by another experimentally demonstrated route [7,40,41]. Because metal–boron bonding can make selective extraction difficult, synthesis, phase purity, surface chemistry, and structural stability remain central research topics [40,41].

### 2.6. Initial Phase (2017–2020): Conceptual Foundations

From 2017 to 2020, annual output in the author-generated dataset rose from one to eight publications. Primary studies from this period covered theoretical prediction of MBene structures and properties, evaluation of layered MAB precursors, and early experimental attempts to remove Al and isolate two-dimensional borides [37,38,40].

### 2.7. Growth and Expansion Phase (2021–2024): Rapid Diversification

Between 2021 and 2024, the number of indexed publications increased from 20 to 76. The literature from this period broadened from structural prediction and synthesis toward energy storage, electrocatalysis, and related functional applications [37,42]. Claims that MBenes are more oxidation-resistant than MXenes remain composition- and condition-dependent and require controlled comparisons [6,8,9,20].

### 2.8. High-Output and the Partial-Year Phase (2025–2026)

The dataset recorded 110 publications in 2025, the highest complete-year count in the analysis. Only three 2026 records were available on 14 April 2026; this partial-year value cannot be interpreted as a decline. The recent literature includes reviews, computational studies, experimental synthesis, and proposed hybrid structures [7,12,13].

### 2.9. Overall Insights

Across the complete years 2017–2025, the dataset shows strong publication growth and a broader range of research themes. The evidence supports a shift from a predominantly predictive literature toward a mixture of computational and experimental work, but it does not establish that MBenes have reached technological or commercial maturity [4,5,6,8,37,43,44,45].

Future work is likely to emphasize reproducible synthesis, controlled surface chemistry, stability, scalable production, and application-specific testing [6,7,12,13,39]. Machine learning–assisted screening and in situ characterization may support these goals, but industrial translation should be discussed as a research objective rather than a forecast [6,7,12,13,39].

In summary, the increase from one indexed publication in 2017 to 110 in 2025 demonstrates rapid growth in research activity. The available evidence, nevertheless, remains uneven: computational and energy-related studies are numerous, whereas direct membrane and desalination studies are limited [8,12,13,37,45].

The trajectory of the field suggests movement from discovery toward synthesis optimization and application testing [3,4,8,12,46]. MBenes may complement other two-dimensional materials, but claims that they will surpass MXenes require composition-specific comparisons under identical conditions [12,46].

The bibliometric mapping indicates substantial participation by Chinese institutions together with contributions from other regions. The dominant themes are computational modeling, experimental synthesis, energy storage, and catalysis. The principal translational needs are reproducible scale-up, phase and surface control, environmental assessment, and standardized application testing [6,7,12,13,39].

For descriptive purposes, the annual publication profile can be divided into an initial phase (2017–2020), an expansion phase (2021–2024), and a high-output complete year in 2025. The partial 2026 count is reported separately and is not treated as evidence of saturation or decline.

### 2.10. Desalination Technologies and Their Challenges

Reverse osmosis generally requires less energy than conventional thermal desalination processes, but its performance remains constrained by pressure requirements, membrane fouling, chemical cleaning, membrane replacement, and brine management [14,15]. Emerging processes, including forward osmosis, membrane distillation, capacitive deionization, electrodialysis, and renewable energy–assisted desalination, may provide energy or operational advantages under specific conditions [18,23]. However, their performance depends strongly on feed salinity, system configuration, operating conditions, energy-recovery arrangements, and the basis used for comparison [47,48].

### 2.11. Membrane Materials: Polysulfone and Its Limitations

The hydrophobicity of polysulfone can promote foulant attachment, pore obstruction, permeability decline, and increased cleaning requirements [17,24,25,26,27]. Hydrophilic coatings, chemical grafting, and the incorporation of nanomaterials such as graphene oxide and metal oxides have, therefore, been investigated to improve membrane wettability and fouling resistance [49,50]. Nevertheless, the magnitude of improvement varies with the nanomaterial properties, loading, fabrication method, membrane structure, feed composition, and operating conditions [24,25,26,27,49,50].

### 2.12. Two-Dimensional (2D) Materials for Membrane Applications

#### 2.12.1. Overview

The isolation of graphene has stimulated broad research into atomically thin materials and their transport properties [9,10,11,12,32]. In membrane systems, nanoporous graphene, graphene oxide laminates, MoS_2_ laminates, and Ti_3_C_2_T_x_ MXene membranes have each demonstrated water or ion transport under specific experimental conditions [9,10,11,12,32,51,52,53,54]. Their mechanisms and performance depend on the pore architecture, interlayer spacing, surface chemistry, support, feed, and operating mode [51,52,53,54].

Two-dimensional materials have been investigated as freestanding layers, laminates, coatings, mixed-matrix fillers, and components of thin-film composites [24,25,26,49,50,55]. Carbonaceous fillers illustrate that oxidation state, surface functional groups, dispersion, loading, and polymer-filler compatibility can alter membrane morphology, wettability, permeability, and fouling behavior [24,25,26]. Agglomeration at unsuitable loadings can instead create defects or obstruct transport pathways [49,50,55].

#### 2.12.2. Graphene and Graphene Oxide (GO)

Graphene is a one-atom-thick carbon lattice, and nanoporous single-layer graphene has demonstrated nearly complete salt exclusion in specialized small-area experiments [10,51,52,53,56,57,58,59]. Graphene oxide (GO) contains oxygen-bearing functional groups that increase its affinity for water and enable lamellar membrane assembly [10,51,52,53]. GO membranes can sieve ions and molecules when their interlayer spacing is controlled, but performance depends on the swelling, functionalization, membrane thickness, defects, and test configuration [56,57,58,59].

GO laminates can swell in water, increasing interlayer spacing and reducing ion selectivity [52,53]. Physical confinement, cross-linking, cation-controlled spacing, and composite architectures have, therefore, been investigated to stabilize transport channels [57,59]. These strategies have produced experimentally measurable ion sieving, but the reported values are specific to the membrane configuration and operating conditions [52,53,57,59].

#### 2.12.3. Transition-Metal Dichalcogenides (TMDs)

TMDs such as MoS_2_ provide chemically and structurally distinct transport channels from graphene-based membranes [11,54]. Functionalized and porous MoS_2_ laminates have demonstrated salt rejection and water transport in forward- and reverse-osmosis experiments [60,61]. For example, a peptide-functionalized porous MoS_2_ laminate maintained more than 95% NaCl rejection during a 30-day forward-osmosis test and exhibited a water permeance of approximately 5 L m^−2^ h^−1^ under the reported conditions [11,54,60,61].

The preparation and scale-up of TMD membranes remain sensitive to the nanosheet production, pore formation, functionalization, laminate assembly, support selection, and control of interlayer spacing [11,54]. Liquid-phase exfoliation and sonication-based processing can support membrane fabrication, but cross-study performance cannot be compared without harmonized feed and operating conditions [60,62].

Accordingly, TMD results are best used as experimentally grounded analogous evidence [61,63]. They demonstrate that surface charge, pore size, interlayer structure, and functionalization can govern permeability and selectivity [61,63].

Silver nanoparticles have been incorporated into PSU ultrafiltration and thin-film composite RO membranes to mitigate bacterial attachment and biofilm development [28,29,30]. These studies support the antimicrobial value of immobilized silver under the tested conditions, while also identifying leaching, long-term activity, and environmental release as considerations for further evaluation [28,29,30].

### 2.13. MBenes: A New Frontier

The evidence in this review is divided into direct experimental MBene studies; computational MBene studies; analogous membrane studies involving MXenes, graphene and GO, and TMDs; and prospective MBene membrane applications [9,10,11,12,20,21]. Ti_3_C_2_T_x_ is an MXene derived from a MAX phase and is not an MBene.

Results from these analogous materials are, therefore, used only to identify mechanisms and design strategies that require subsequent validation in true MBene membranes [35,57,64,65].

As we can in Table 1, MBenes are an emerging family of two-dimensional transition-metal borides associated with layered MAB precursors [3,4,5,37]. Experimental and computational studies report composition-dependent electronic, structural, catalytic, adsorption, and electrochemical properties, yet the material-level results of these studies do not establish pressure-driven membrane permeability, salt rejection, fouling resistance, or durability [8,42,46,66]. Direct water-related evidence now includes MBene-based solar interfacial evaporators reported in 2024, including layered membranes, polymer-supported aerogels, and rGO-MBene monoliths [12,13,43]. These studies demonstrate photothermal freshwater production under solar evaporation conditions, but they are not equivalent to pressure-driven RO, NF, or UF membrane tests [12,13,43]. Ti_3_C_2_T_x_ MXenes have been tested directly as lamellar ion-sieving and antibacterial membranes, and are therefore used only as benchmarks [9,21,22].

Challenges relevant to future MBene-polymer membranes include precursor availability, selective extraction, phase purity, surface termination control, oxidation or hydrolysis, nanosheet dispersion, aggregation, interfacial compatibility, leaching, and reproducible scale-up [6,7,9,22]. Nonetheless, analogous MXene and polymer-nanocomposite studies provide strategies for intercalation, surface modification, and dispersion control [25,38,39,49,50].

Our analysis shows that most MBene studies address energy storage, catalysis, supercapacitors, synthesis, and DFT modeling. Pressure-driven water-treatment and desalination membranes remain comparatively underexplored. Experimental MBene-based solar evaporation and water-purification systems are nevertheless reported [12,13,43]. Future work should distinguish solar evaporators from selective filtration membranes and should prioritize standardized permeability, rejection, fouling, stability, leaching, and pilot-scale testing [6,7,12,13,39]. Selected MXene reviews are retained only for broad comparison and are supplemented throughout by original experimental studies [33,34,64,65].

### 2.14. Research Gap: Why MBenes Remain Underexplored in Water Treatment

The current literature is dominated by batteries, supercapacitors, electrocatalysis, synthesis, and DFT modeling. Direct MBene-based solar evaporation and water-purification studies exist, but pressure-driven desalination and membrane-filtration evidence remains limited [12,13,43]. Long-term pressure-driven fouling resistance, salt rejection, and structural stability, therefore, remain insufficiently established [12,13,43]. This gap supports future MBene-PSU research under controlled filtration conditions [12,13,43,49,50].

To understand why this specific gap in pressure-driven membrane applications persists, it is necessary to examine the broader thematic focus of the field. The keyword co-occurrence network analyzed in the following section illustrates how theoretical modeling, catalysis, and energy storage have dominated the initial wave of MBene research, providing context for why water-treatment applications remain an underexplored frontier.

## 3. Keyword Landscape and Research Focus

**Figure 2:**
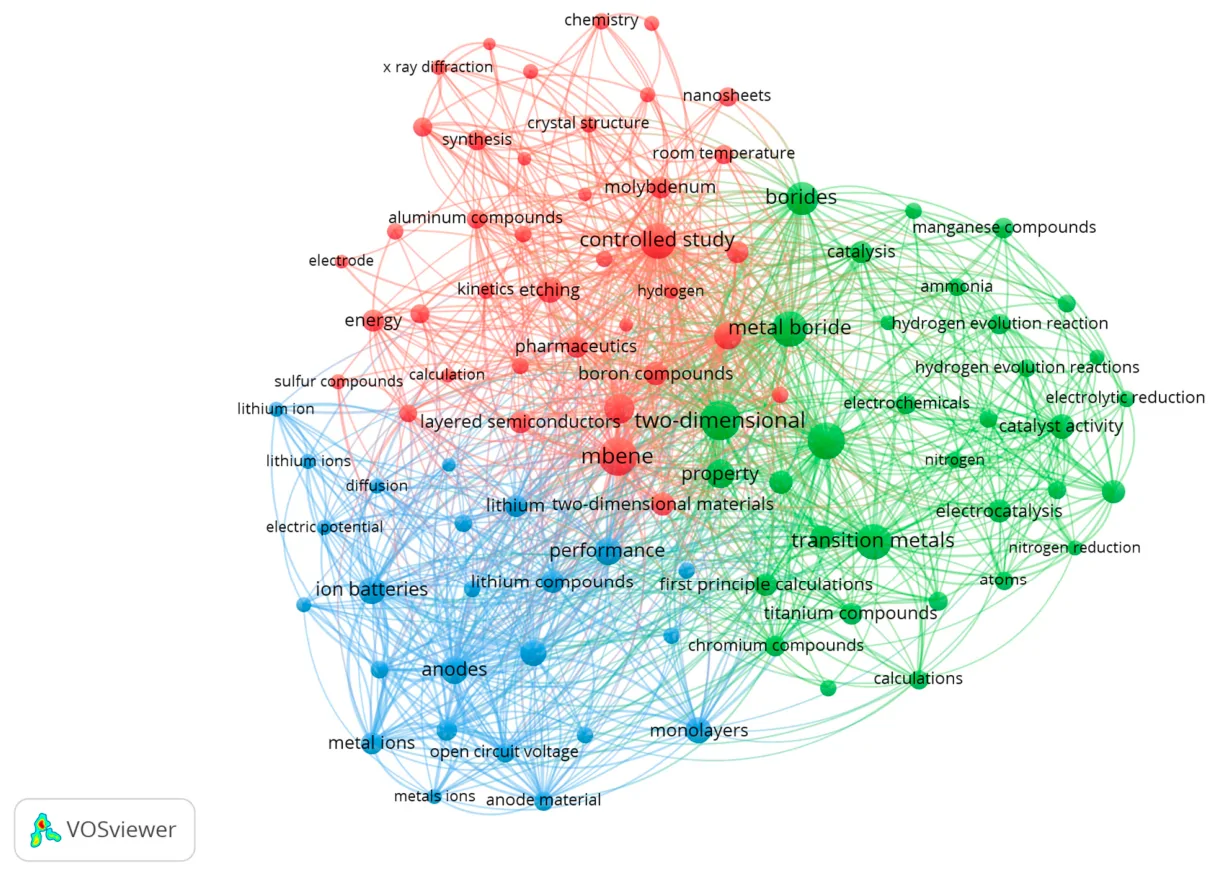
Keyword co-occurrence network generated by the authors in VOSviewer from 279 Scopus records retrieved on 14 April 2026; data processing and network settings are reported in Section 2.

Figure 2 shows that frequent terms include “two-dimensional,” “MBene,” “density functional theory,” and “transition metals.” The co-occurrence of computational terms with application terms related to batteries, electrocatalysis, and adsorption indicates that the retrieved literature combines predictive modeling with a growing body of experimental and application-oriented work. This interpretation is descriptive of the author-generated network and does not establish application readiness [36].

1. Geographic and Institutional Contributions

In the author-generated dataset, China was associated with 205 publications, followed by the United States (28), Germany (17), India (16), and Turkey (12). The National Natural Science Foundation of China was listed in 139 records. Frequently represented affiliations included the Ministry of Education of the People’s Republic of China (32 records), the Chinese Academy of Sciences (16), Hebei University of Technology (13), Beihang University (11), and City University of Hong Kong (11). These counts describe Scopus affiliation and funding fields; they do not measure research quality or causal effects of funding.

2. Research Themes and Applications

The thematic scope of MBene research can be broadly categorized into synthesis, electronic modeling, and functional applications [3,4,5,6,8]. Early studies concentrated on structural derivation from MAB phases, exploring etching methods to remove A-layer atoms and produce stable boride monolayers [37,46]. The focus has since shifted toward electronic property engineering through DFT-based calculations that analyze band structures, charge density, and adsorption energies [3,4,5,6,8,37,46]. These studies lay the groundwork for applications in electrocatalysis and energy storage [3,4,5,6].

The literature and keyword network emphasize predicted or experimentally investigated roles in batteries, supercapacitors, and electrocatalysis [8,42]. Terms associated with lithium- and sodium-ion storage, hydrogen evolution, nitrogen reduction, adsorption, and heterostructures indicate broad application screening [67,68,69]. Many reported properties remain computational, so application claims should be identified as predicted unless an experimental study is cited [8,42,45,67,68,69].

### 3.1. Cluster List

This bibliometric mapping reveals three dominant clusters:

- Cluster 1 (Synthesis, Structure, and Characterization): Focused on material preparation, structural properties, and experimental validation.
- Cluster 2 (Catalysis and Electronic Mechanisms): Centered on density functional theory (DFT) modeling, adsorption behavior, and catalytic activity.
- Cluster 3 (Electrochemical Applications and Energy Storage): Encompassing lithium/sodium-ion batteries, diffusion barriers, and electrode performance.

Together, these clusters outline a rapidly growing multidisciplinary field combining computational chemistry, materials engineering, and electrochemical energy systems.

### 3.2. Cluster 1: Synthesis, Structural Characterization, and Fundamental Properties

Cluster 1 contains key terms such as “controlled study,” “etching,” “scanning electron microscopy,” “X-ray diffraction,” “molybdenum compounds,” “aluminum,” “boron,” and “two-dimensional materials.” These reflect the experimental foundation of MBene research.

The keyword “etching” occurred 28 times and had a total link strength of 225 in the author-generated network [3,5,6,7,38,39]. MBene synthesis commonly investigates selective removal of the A-layer from MAB phases, but strong metal–boron bonding can require chemistries different from conventional MXene etching [3,5,6,7]. Reported approaches include molten-salt, halogen-assisted, and related extraction routes, with resulting surface chemistry dependent on the composition and process [38,39].

The occurrence of X-ray diffraction, X-ray photoelectron spectroscopy, scanning electron microscopy, and related terms reflects the importance of phase, morphology, bonding, and surface-chemistry characterization when distinguishing precursors, reaction products, and two-dimensional borides [4,6,7,39].

The keywords “crystal structure” and “layered semiconductors” indicate continued attention to stacking, bonding, electronic structure, and ion transport–relevant geometry [46,66,70,71]. The properties are composition-specific and should not be generalized across the entire MBene family [46,66,70,71].

Keywords referring to molybdenum, aluminum, boron, titanium, and other elements indicate compositional screening across multiple precursors and MBene candidates [37,46,72,73,74]. Reported conductivity, stability, and catalytic behavior vary with composition, structure, defects, and surface chemistry [37,46,72,73,74].

The terms “surface property” and “nanomaterial” are consistent with interest in oxidation, termination chemistry, heteroatom incorporation, and composite formation [39,75,76,77]. These strategies are investigated to tune electronic and adsorption behavior, but their effects require material-specific validation [39,75,76,77].

The index term “controlled study” occurred 53 times and had a total link strength of 573. Because this is a database-indexing term, its frequency should not be interpreted as proof of experimental reproducibility. Reproducibility must instead be assessed from reported synthesis conditions, characterization, controls, and independent replication.

Hence, Cluster 1 represents the materials science core of MBene research—devoted to synthetic innovation, morphological verification, and establishing baseline physical–chemical properties [4,5,6,7,39].

### 3.3. Cluster 2: Catalytic Mechanisms, Electronic Properties, and Theoretical Modeling

Cluster 2 is dominated by terms including “density functional theory,” “transition metals,” “catalyst activity,” “adsorption,” “hydrogen evolution reaction,” and “electronic structure,” indicating that theoretical and computational chemistry form a major part of the retrieved MBene literature.

The term “density functional theory” occurred 58 times and had a total link strength of 492 in the author-generated network. DFT studies calculate formation energies, electronic structures, adsorption energies, and charge-transfer behavior to screen candidate MBenes before or alongside experimental work [37,42,78].

The frequent appearance of “calculations,” “free energy,” and “electronic structure” further reflects thermodynamic and electronic modeling across MBene candidates. These results remain dependent on the selected structure, function, boundary conditions, and model assumptions [46,79,80].

Keywords for catalysis, hydrogen evolution, and nitrogen reduction show that MBenes are widely screened for HER and NRR [42,81,82,83,84]. The evidence includes a large computational component, so catalytic promise should not be presented as experimentally established for the entire material family [42,81,82,83,84].

Computational studies evaluate MBenes as possible non-noble-metal catalysts by examining electronic structure and adsorption energetics [68,78]. Cost, abundance, catalytic activity, selectivity, and stability must nevertheless be demonstrated for each composition and operating environment [85,86,87].

The co-occurrence of ammonia, hydrogen, HER, and NRR terms indicates that several MBene compositions have been screened for hydrogen-evolution and nitrogen-reduction pathways. Much of this evidence is computational rather than device-level experimental validation [42,81,82,84].

Keywords related to adsorption, gas adsorption, charge transfer, and electrocatalysis reflect modeling of surface–adsorbate interactions [76,80]. Reported adsorption energies and charge-transfer mechanisms are composition- and model-dependent and should not be treated as universal MBene properties [88,89,90,91,92].

Transition-metal identity can alter the electronic states involved in adsorption and catalysis [46,72]. The network, therefore, supports compositional diversity as a research theme, while quantitative catalytic comparisons require source-specific calculations or experiments [77,85].

Cluster 2 contains terms associated with chromium, iron, manganese, molybdenum, and titanium compounds, reflecting the computational comparison of multiple transition-metal-boride candidates. Representative themes include:

- Ti-Containing MBene Candidates: Electronic structure and mechanical-property screening [37,46,87].
- Mo-Containing MBene Candidates: Electronic and catalytic-property screening [68,69,93].
- Cr-Containing MBene Candidates: Adsorption and catalytic-property screening [40,81,86].

This compositional diversity supports systematic candidate screening, while source-specific evidence is required for any quantitative comparison [37,46,74,94].

Terms related to electronic properties, conductance, and electric potential show that charge transport is an important research theme [46,72,73,95]. DFT predicts metallic or semi-metallic behavior for selected structures, but experimental conductivity and device performance must be reported for each material [46,72,73,95].

Electronic structure influences calculated adsorption and ion-migration energetics [42,78,79,83]. The magnitude and practical significance of these relationships are composition- and model-specific [42,78,79,83].

The simultaneous presence of theoretical and experimental terms indicates interaction between prediction, synthesis, and characterization [5,6,7,37,46]. Keyword co-occurrence does not establish that a particular DFT prediction resulted in successful synthesis unless the original study documents that link [5,6,7,37,46].

In summary, Cluster 2 represents theoretical and mechanistic work used to explain and predict MBene electronic, adsorption, and catalytic behavior [42,46,85,90].

### 3.4. Cluster 3: Electrochemical and Energy Storage Applications

Cluster 3 contains terms including “anodes,” “lithium-ion batteries,” “sodium-ion batteries,” “diffusion barriers,” “electrochemical performance,” and “supercapacitor,” representing the energy-storage portion of the retrieved literature.

Anode- and electrode-related keywords show that selected MBenes are investigated as rechargeable-battery candidates [8,44,45,67]. Conductivity, ion-accessible structure, and mechanical response are evaluated as possible advantages, but high capacity and fast charging must be demonstrated for each composition [8,44,45,67].

The occurrence of lithium-, sodium-, potassium-, and metal-ion terms indicates multi-ion screening [37,74]. Computational studies commonly evaluate adsorption sites, migration pathways, and open-circuit voltages, whereas experimental validation remains more limited [79,96,97].

The prominence of lithium-ion batteries, sodium-ion batteries, and diffusion barriers reflects computational and experimental evaluation of ion storage and migration. Numerical diffusion barriers vary with composition, termination, coverage, and computational method and are, therefore, not summarized as a single range [74,79,96,97].

Predicted open-circuit voltages and theoretical capacities also vary substantially among MBene compositions and adsorption models [79,96,97,98,99]. These values should be reported only in direct association with the original study and should not be used to claim general superiority over MXenes [79,96,97,98,99].

The terms “electrochemical performance,” “diffusion,” and “cycling” show that both modeling and experimental studies evaluate ion transport, capacity retention, and structural response. Strong metal–boron bonding may contribute to stability in some structures, but cycling behavior must be demonstrated experimentally for each electrode [8,45,100].

Cyclic voltammetry, galvanostatic charge–discharge testing, and electrochemical impedance spectroscopy are used in experimental electrode studies [8,44,45,67]. Their presence does not imply that every MBene composition has been experimentally validated [8,44,45,67].

The keyword “functionalized” indicates investigation of surface modification, doping, and hybridization with conductive or stabilizing components [75,97,100,101]. Reported benefits remain dependent on composition, synthesis, and test conditions [75,97,100,101].

The occurrence of “supercapacitor,” “conductance,” and “energy storage” shows interest beyond batteries. Claims concerning high-power performance or wide operating windows require direct electrochemical measurements and should not be inferred from metallic conductivity alone [44,45,67].

Diffusion barriers and ion-transport pathways are commonly studied using DFT-based and molecular-simulation methods [79,96,97,98]. These calculations can guide candidate selection, but they do not replace experimental measurement of transport, cycling, and stability [79,96,97,98].

### 3.5. Inter-Cluster Relationships and Cross-Domain Integration

The three clusters—synthesis (C1), theory (C2), and application (C3)—are tightly interconnected, forming a looped innovation cycle typical of emerging materials:

1. Synthesis (C1) develops new MBenes with tailored morphology and purity;
2. Theory (C2) predicts catalytic and electronic properties via DFT, identifying promising compositions;
3. Applications (C3) test these materials in batteries, supercapacitors, and electrocatalytic setups;
4. The performance feedback then informs new synthesis modifications, closing the cycle.

The relationship among synthesis, modeling, and application testing is iterative: computational predictions guide candidate selection, characterization tests phase and structure, and application results reveal which predicted properties persist in real materials [5,6,8,37,45]. The bibliometric co-occurrence network supports this general interaction but does not establish a specific synthesis–property mechanism [5,6,8,37,45].

The overlap of catalytic and electrochemical terms indicates that some MBene compositions are investigated for more than one application [8,67,69]. Such multifunctionality remains composition- and evidence-specific and should be distinguished between computational prediction and experimental demonstration [8,67,69].

The appearance of terms such as “pharmaceutics” and “unclassified drug” may reflect emerging biomedical or environmental indexing, but keyword occurrence alone does not demonstrate biocompatibility, drug-delivery performance, or pollutant-removal efficacy. These applications remain exploratory [76,102,103,104].

### 3.6. Emerging Trends and Research Gaps

The dataset is concentrated in Ti-, Mo-, and Cr-associated terms, while zinc-, manganese-, and iron-related terms occur less frequently. This pattern supports broader compositional screening as a research opportunity, not a prediction of superior performance.

Compared with the extensively studied termination chemistry of MXenes, experimental control of MBene surface chemistry remains less developed [6,7,38,39]. Chlorine- and sulfur-related terms indicate interest in alternative synthesis or functionalization routes, but selectivity and stability benefits require direct measurement [6,7,38,39].

Developing lower-hazard, lower-temperature, and scalable synthesis routes is an important research objective [5,7,39]. The occurrence of terms such as “room temperature” does not by itself demonstrate reduced energy consumption, environmental impact, or commercial feasibility [5,7,39].

Co-occurrence among terms for nanosheets, monolayers, graphene, and MXenes indicates interest in hybrid or heterostructured materials [69,75,100,101]. Any claimed mechanical, transport, or electrochemical synergy must be validated in a defined composition and test system [69,75,100,101].

Machine learning–assisted screening and in situ characterization could accelerate candidate selection and synthesis optimization [3,46,66,105]. These approaches are presented as future research directions rather than established trends unless supported by dedicated studies [3,46,66,105].

The limited prominence of scale-up and device-fabrication terms is consistent with a translational gap [6,7,12]. Pilot-scale synthesis, batch-to-batch reproducibility, application-specific integration, and environmental assessment are needed before industrial relevance can be established [13,39,43].

### 3.7. Hypothesized Advantages of MBenes and Requirements for Validation

Selected MBenes exhibit or are predicted to exhibit material-level characteristics potentially relevant to membrane development, including metal–boron bonding, electrical conductivity, and tunable composition or surface chemistry [8,39]. These properties have not been translated into experimentally verified superiority over MXenes or other two-dimensional desalination membranes [46,72,73].

Possible oxidation resistance, saline stability, reduced degradation, and lifecycle benefits should, therefore, be treated as hypotheses [9,10,11,12,13]. Validation requires direct, controlled comparisons of MBenes, MXenes, and other membrane nanomaterials under identical fabrication and operating conditions, including permeability, selectivity, fouling, oxidation, leaching, membrane lifetime, scalability, environmental impacts, and lifecycle cost [9,10,11,12,13,43].

### 3.8. Conceptual Framework Derived from the Bibliometric Network

The following conceptual flow can be derived:

1. Material Genesis: MAB → MBene (etching and structural verification).
2. Theoretical Screening: DFT predicts properties (band structure and adsorption energy).
3. Functional Evaluation: Catalysis (HER/NRR) and energy storage (Li/Na-ion).
4. Performance Optimization: Surface functionalization, doping, and hybridization.
5. Application Diversification: Batteries, supercapacitors, sensors, possibly biomedical uses.

This pipeline reflects an integrated research ecosystem, where computational modeling, materials chemistry, and electrochemical engineering converge [5,8,42].

### 3.9. Mapping the Future of MBene Research

The bibliometric dataset reveals that MBene research has evolved from exploratory synthesis to a well-defined multidisciplinary domain encompassing theoretical modeling, catalytic applications, and energy storage technologies. The strong co-occurrence of “density functional theory,” “transition metals,” “etching,” and “anodes” indicates a dynamic interplay between computation, synthesis, and application [42,45].

- Cluster 1 (Experimental Core): Focuses on developing reproducible synthesis and structural validation of MBenes using advanced microscopy and spectroscopy [4,5,6,7,39].
- Cluster 2 (Theoretical Insights): Uses DFT and first-principle methods to explore adsorption, charge transfer, and catalytic activity for hydrogen and nitrogen reactions [92,105,106].
- Cluster 3 (Applications Frontier): Covers predicted and experimentally investigated roles in lithium- and sodium-ion batteries, supercapacitors, and other electrochemical systems [8,44,45,67,100].

## 4. Comparative Analysis

Direct performance comparisons between MBenes and established two-dimensional pressure-driven membranes remain premature because the direct MBene water literature currently concerns solar interfacial evaporation rather than comparable RO, NF, or UF testing [9,10,11,12,13,22]. Available graphene and graphene-oxide, transition-metal-dichalcogenide, and MXene studies also use different membrane configurations, feeds, pressures, temperatures, durations, and performance indicators [52,53,54]. The comparisons in this section, therefore, map material characteristics and research opportunities rather than establish MBene superiority [56,57,60].

Graphene and graphene oxide, transition-metal dichalcogenides, and MXenes have demonstrated permeability, selectivity, or fouling-related behavior in experimental membrane systems [9,10,11,12,13,21], as shown Table 2. These findings provide analogous evidence for future MBene filtration-membrane design but do not validate equivalent MBene performance [43,57,60,107,108]. Direct MBene solar-evaporation studies demonstrate photothermal freshwater production, whereas pressure-driven MBene permeability, salt rejection, fouling resistance, antimicrobial activity, and long-term durability remain insufficiently established [60,107,108].

### 4.1. Synthesis and Properties of MBenes

MBenes are associated with layered MAB precursors and two-dimensional transition-metal-boride products [3,4,5,6,7]. Selective removal of the A-layer is a central route, while experimental reports also illustrate that the attainable structure depends strongly on precursor chemistry and extraction conditions [3,4,5,6,7,38,39,40]. Strong metal–boron bonding, precursor availability, phase purity, and termination control make MBene preparation challenging [38,39,40]. Molten-salt and halogen-assisted routes are among the approaches discussed in the literature, but scalable production with reproducible phase and surface chemistry remains to be demonstrated [3,4,5,6,7,38,39,40].

Material-level experiments and calculations describe composition-dependent conductivity, mechanical behavior, adsorption, and surface chemistry that could be relevant to separation materials [8,46,66]. However, hydrophilicity, surface termination density, specific surface area, mechanical strength, and chemical stability cannot be assigned universal values across the MBene family [72,73]. Their behavior in saline water and polymeric membranes, therefore, requires direct characterization [8,46,66,72,73].

Doping, functionalization, and alternative extraction methods have been proposed to tune MBene properties, but the cited literature does not establish general percentage improvements in heavy-metal adsorption, synthesis yield, or energy use [39,75,76,97]. Future membrane studies should report precursor conversion, product purity, surface composition, yield, batch reproducibility, and stability together with application performance [39,75,76,97].

### 4.2. Incorporating MBenes into Polysulfone Membranes

Direct experimental MBene water studies currently concern solar interfacial evaporation and related photothermal purification rather than pressure-driven MBene-PSU desalination membranes [12,13,43]. MXene studies, nevertheless, provide analogous guidance on hydrophilic surface chemistry, interlayer transport, membrane assembly, and antibacterial behavior [9,21,22,64,65].

For future MBene-PSU studies, phase inversion, surface coating, and interfacial assembly are plausible fabrication routes [24,25,26]. Established PSU and PES modification studies show that nanofiller loading, dispersion, polymer–filler interactions, and membrane morphology can alter permeability and fouling resistance [24,25,26,49,50,55]. Experimental design and multi-objective optimization may support formulation development, but no verified MBene-specific reductions in fouling, cost, energy use, or lifecycle impact can yet be claimed [49,50,55].

Aggregation, non-selective defects, pore blockage, leaching, and changes in mechanical integrity are potential risks when incorporating nanosheets into polymers [24,25,26]. Surface functionalization or hybrid fillers may improve dispersion in some nanocomposite systems, but the optimal loading and performance must be determined experimentally for each MBene composition and membrane process [49,50,55].

### 4.3. Fouling and Antifouling Properties

Membrane fouling reduces permeability, increases cleaning demand, and can shorten operating life [17,21]. Hydrophobic PSU and PES membranes are particularly susceptible to adsorption of organic and biological foulants, so hydrophilic and nanomaterial-based modifications are widely investigated [23,24,25,49,50]. Ti_3_C_2_T_x_ MXene membranes have demonstrated antibacterial activity against Escherichia coli and Bacillus subtilis [49,50]. Direct MBene tests should quantify foulant adsorption, flux decline and recovery, cleaning response, microbial attachment, and material release [17,21].

Photocatalytic, electrothermal, and hybrid-nanomaterial self-cleaning concepts have been investigated across the wider membrane literature [12,13,32]. The references cited here do not support specific MBene-TiO_2_, MBene-ZnO, or MBene-graphene removal percentages or membrane-lifetime extensions [43,55,64,65]. Such systems should, therefore, be treated as prospective designs requiring controlled irradiation or electrical input, mass-balance analysis, reuse testing, and assessment of nanomaterial stability [55,64,65].

Natural organic matter can adsorb on membrane surfaces and within pores, causing flux decline and irreversible fouling [17,24,25,26]. Hydrophilic surfaces and appropriately dispersed nanofillers can reduce foulant interactions, but results depend on filler chemistry, loading, membrane morphology, and feed conditions [49,50]. These general nanocomposite findings provide design context for future MBene membranes rather than direct MBene evidence [49,50].

### 4.4. Energy Efficiency and Sustainability

Energy consumption is a central consideration in desalination [14,47,48]. Reviews of seawater RO report desalination-stage-specific energy consumption commonly in the approximate range of 2.5–4.0 kWh m^−3^, with plant-level values depending on feed salinity, pre-treatment, post-treatment, recovery, pumping, and energy-recovery design [14,47,48]. More permeable or fouling-resistant membranes may reduce pressure or cleaning requirements, but no source identified in this review verifies a specific energy or carbon-emission saving for an MBene-PSU membrane [14,47,48].

Lifecycle and sustainability claims require system boundaries that include precursor production, etchants, solvents, membrane fabrication, operating energy, cleaning, replacement, material release, brine management, and end-of-life treatment [2,14,16,18]. The reviewed literature does not provide a comparative lifecycle assessment of MBene membranes [18,47,48]. Consequently, percentage reductions in environmental impact, waste, or ecological damage have been removed and should remain unclaimed until supported by a transparent lifecycle inventory [47,48].

### 4.5. Applications Beyond Desalination

Primary MBene studies report solar interfacial evaporation, adsorption-assisted removal of selected metal ions, photocatalytic functions, and sensing, while computational studies examine gas capture and catalytic conversion [12,13,43,76]. These results are composition- and configuration-specific and do not establish general membrane efficiencies for dyes, metals, oil–water mixtures, or gases [76,80,89,103]. Application claims must, therefore, remain linked to the tested material, process, and operating conditions [80,89,103].

Other polymer nanocomposites, including carbon- and metal oxide–containing membranes, demonstrate that hydrophilicity, photocatalytic activity, porosity, and dispersion can influence permeability, fouling, and solute removal [49,50,55]. These systems provide analogous design principles only; their performance cannot be assigned to MBenes without direct comparative experiments [49,50,55].

Emerging contaminants and air-filtration particles require compound-specific adsorption, degradation, or rejection tests [9,12,13,103]. Future studies should report contaminant identity and concentration, contact time, water chemistry, mass balance, regeneration, material leaching, and toxicity before claiming broad environmental performance [43,103].

### 4.6. Economic Feasibility and Scalability

The commercial feasibility of MBene-based membranes will depend on precursor availability, synthesis yield and purity, reagent recovery, worker and environmental safety, batch reproducibility, membrane fabrication, service life, and verified performance benefits [6,7,12,13]. The reviewed sources do not substantiate specific cost reductions, production capacities, energy savings for reactors, or three-to-five-year investment payback periods [6,7,12,13]. Techno-economic analysis should, therefore, be deferred until experimentally validated process and membrane data are available [6,7,12,13].

No evidence identified in the cited literature establishes industrial MBene membrane partnerships, a fixed commercialization date, large-scale precursor recycling rates, or a quantified reduction in RO operating expenditure [6,7,8,12]. Future scale-up studies should report material and energy balances, reagent recovery, quality control, waste treatment, membrane manufacturing compatibility, and uncertainty ranges for capital and operating costs [13,43].

## 5. Integrated Evidence Synthesis and Translational Roadmap

### 5.1. Evidence Maturity and Research Gaps

**Figure 3:**
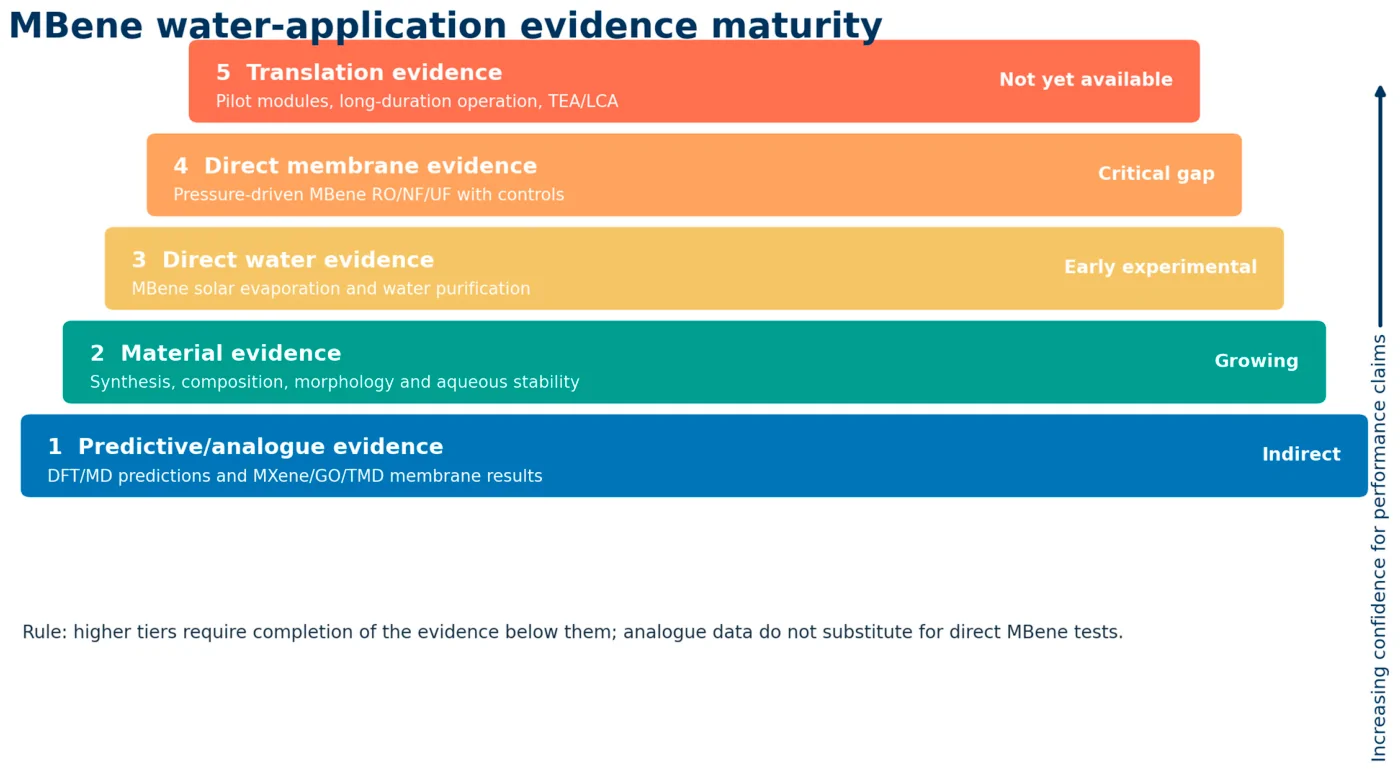
Evidence-maturity hierarchy for MBene water applications. The conceptual synthesis was developed by the authors from primary MBene synthesis studies, two-dimensional membrane experiments, and membrane-evaluation guidance [109,110,111,112,113,114,115,116,117,118,119,120,121].

Figure 3 organizes the literature by the confidence that each evidence type can provide for water-treatment claims. At the base, computational studies and results from MXene, graphene, GO, and TMD membranes can identify plausible transport mechanisms and useful design variables, but they do not demonstrate MBene membrane performance. Molecular simulation of nanoporous graphene, for example, established how pore size and chemistry may govern water-salt selectivity, whereas MXene experiments showed that stacked two-dimensional channels can exhibit charge- and size-dependent ion transport [109,110,111,112,113,114,115,116,117]. These studies justify hypotheses and experimental choices, not numerical transfer to MBenes. The next level is MBene material evidence: controlled precursor conversion, phase identification, exfoliation, composition, and surface characterization. Topochemical, decomposition-based, gaseous-HCl, and alkaline-hydrothermal routes demonstrate that synthesis history strongly affects the resulting boride structure and chemistry [109,110,111,112]. Direct MBene water evidence is presently narrower and should be treated as application-specific rather than universal [118,119,120,121]. A further tier requires an MBene-containing membrane tested under a defined pressure-driven configuration with an appropriate unmodified control, replicated measurements, and complete feed and operating conditions. No analogous experiment can fill that tier. The highest level adds long-duration module operation, cleaning recovery, safety, techno-economic analysis, and life-cycle assessment. Figure 3, therefore, prevents scope conflation: every claim should be positioned at the highest tier directly supported by its source. Advancement is cumulative; a promising calculation does not replace material verification, and a successful bench coupon does not establish pilot readiness [118,119,120].

Table 3 converts the hierarchy in Figure 3 into rules for interpreting the reviewed evidence. The first distinction is between a material being an MBene and a device being an MBene membrane. Experimental formation or exfoliation of a molybdenum boride nanosheet supports statements about synthesis, crystal structure, morphology, and measured surface composition; it does not by itself support claims about salt rejection, permeability, fouling resistance, or module durability [109,110,111,112]. The second distinction concerns application configuration. Solar interfacial evaporation and pressure-driven reverse osmosis, nanofiltration, or ultrafiltration have different driving forces, mass-transfer resistances, performance indicators, and failure modes. Evidence obtained in one configuration should, therefore, remain labeled with that configuration. The third distinction is between prediction and observation. Atomistic calculations can reveal candidate pores, energy barriers, or surface interactions, as illustrated by nanoporous-graphene modeling, but calculated transport must be validated using experimentally realized chemistry and defects [117]. Analogous studies remain valuable because MXene membranes experimentally demonstrate that nanosheet stacking, channel hydration, surface charge, and modification can alter molecular and ionic transport [113,114,115,116]. Their proper role is to identify controls and variables for future MBene experiments. Finally, Table 3 treats absent evidence as a reportable result. If no pressure-driven MBene dataset satisfies minimum identity and test criteria, the defensible conclusion is that performance is undetermined. This evidence discipline allows the review to be forward-looking without converting research opportunities into established advantages, and provides readers with a transparent basis for judging every comparative statement [118,119,120].

### 5.2. From MBene Synthesis to Membrane Testing

Table 4 links each preparation stage to the measurements needed before membrane data can be interpreted. MBenes are not a single invariant filler: the parent phase, conversion pathway, reagent environment, temperature history, washing, and delamination can change phase purity, residual aluminum, oxidation, flake dimensions, porosity, and surface chemistry. Multistep topochemical conversion and controlled precursor decomposition produce distinct structures and intermediates, while gaseous-HCl and alkaline-hydrothermal approaches introduce different process and safety considerations [109,110,111,112]. X-ray diffraction should establish phases; spectroscopy and elemental analysis should examine composition and terminations; microscopy should determine morphology and thickness; and mass balance should document yield and removed species. Once the material is incorporated into a membrane, support identity, fabrication route, loading, active-layer thickness, wet-state spacing, and visible defects become additional causal variables. MXene studies demonstrate that nanosheet assembly, surface control, and channel modification can strongly alter transport, so these variables must be measured rather than assumed for MBenes [113,114,115,116]. Performance testing then requires the exact feed composition, concentration, pH, temperature, pressure or osmotic gradient, cross-flow condition, active area, stabilization time, duration, and cleaning protocol. At least one support or polymer blank prepared by the same procedure is required to attribute any change to the MBene. Replicate membranes and uncertainty estimates distinguish reproducible effects from coupon variability. Table 4, therefore, turns synthesis characterization and membrane testing into one traceable chain, reducing the risk that impurities, processing changes, or uncontrolled defects are misidentified as intrinsic MBene performance [118,120].

**Figure 4:**
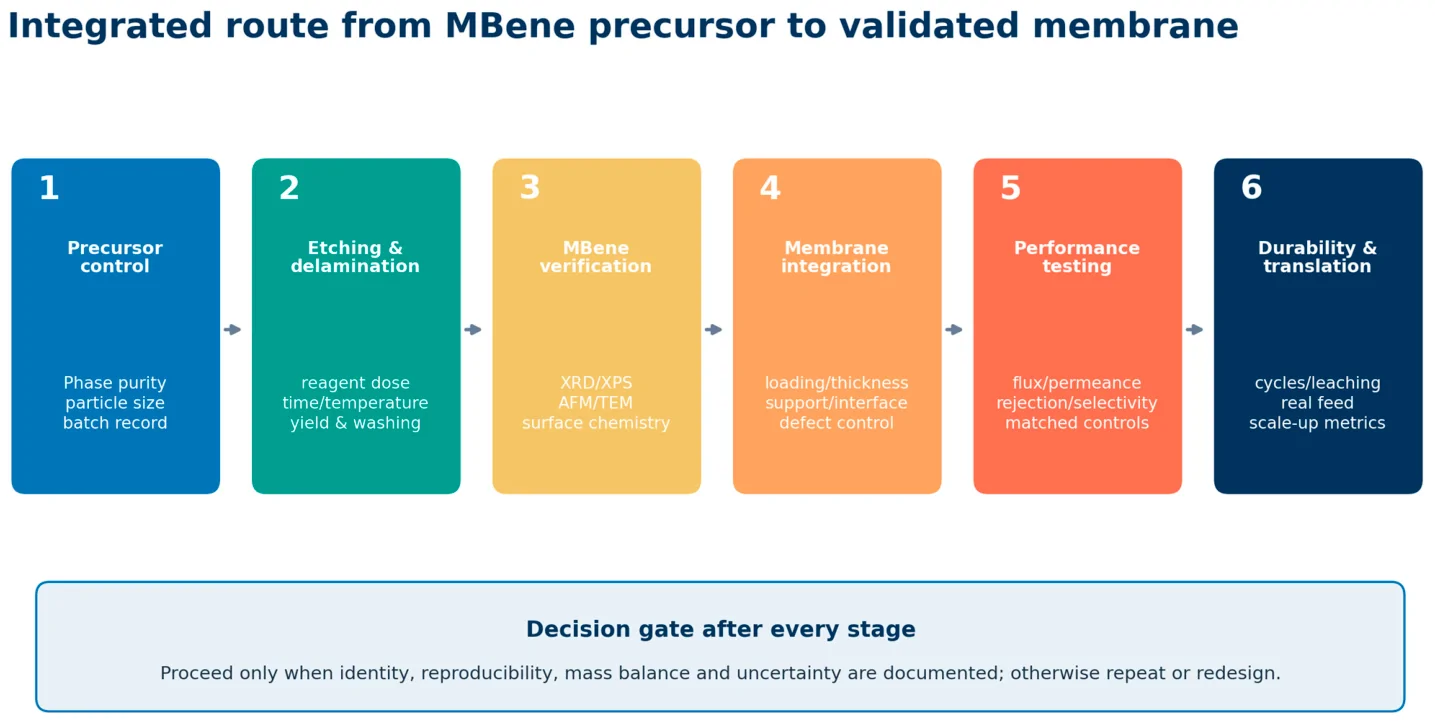
Integrated synthesis–fabrication–characterization–testing workflow for MBene membranes. Each decision gate requires documented identity, reproducibility, and uncertainty before progression [109,110,111,112,113,114,115,116,117,118,119,120].

Figure 4 presents a closed, six-stage workflow rather than a one-way fabrication recipe. Precursor control comes first because phase impurities and particle-size variation can propagate into incomplete conversion and inconsistent nanosheets. The selected etching or conversion route must then be documented through reagent amounts, solid-to-liquid ratio, temperature, time, atmosphere, washing, delamination, and recovered yield. Studies of Mo-Al-B precursors show that low-temperature topochemical transformation, controlled decomposition, gaseous-HCl treatment, and alkaline hydrothermal etching can generate different products and intermediates [109,110,111,112]. The third stage verifies that the intended MBene was actually obtained. Complementary diffraction, spectroscopy, microscopy, thickness, and composition measurements are required because no single technique establishes phase, morphology, and surface chemistry simultaneously. Membrane integration follows only after this identity gate. Fabrication records should connect nanosheet size and concentration to membrane loading, thickness, support, interfacial adhesion, wet-state channel structure, and defect density. MXene membrane studies demonstrate why this gate matters: assembly microstructure and channel modification can determine observed water or ion transport [113,114,115,116]. The fifth stage applies matched controls and standardized conditions to quantify flux or permeance, rejection or selectivity, uncertainty, and mass balance. The final stage challenges the membrane through repeated cycles, cleaning, realistic feeds, leaching measurements, and scale-up analysis. A failed gate sends the study back to the relevant earlier stage; it should not be hidden by reporting only the best coupon. Thus, Figure 4 makes reproducibility a design variable and ensures that a performance value remains traceable to a defined material, membrane, and test protocol [118,120].

### 5.3. Standardized Validation and Practical Translation

**Figure 5:**
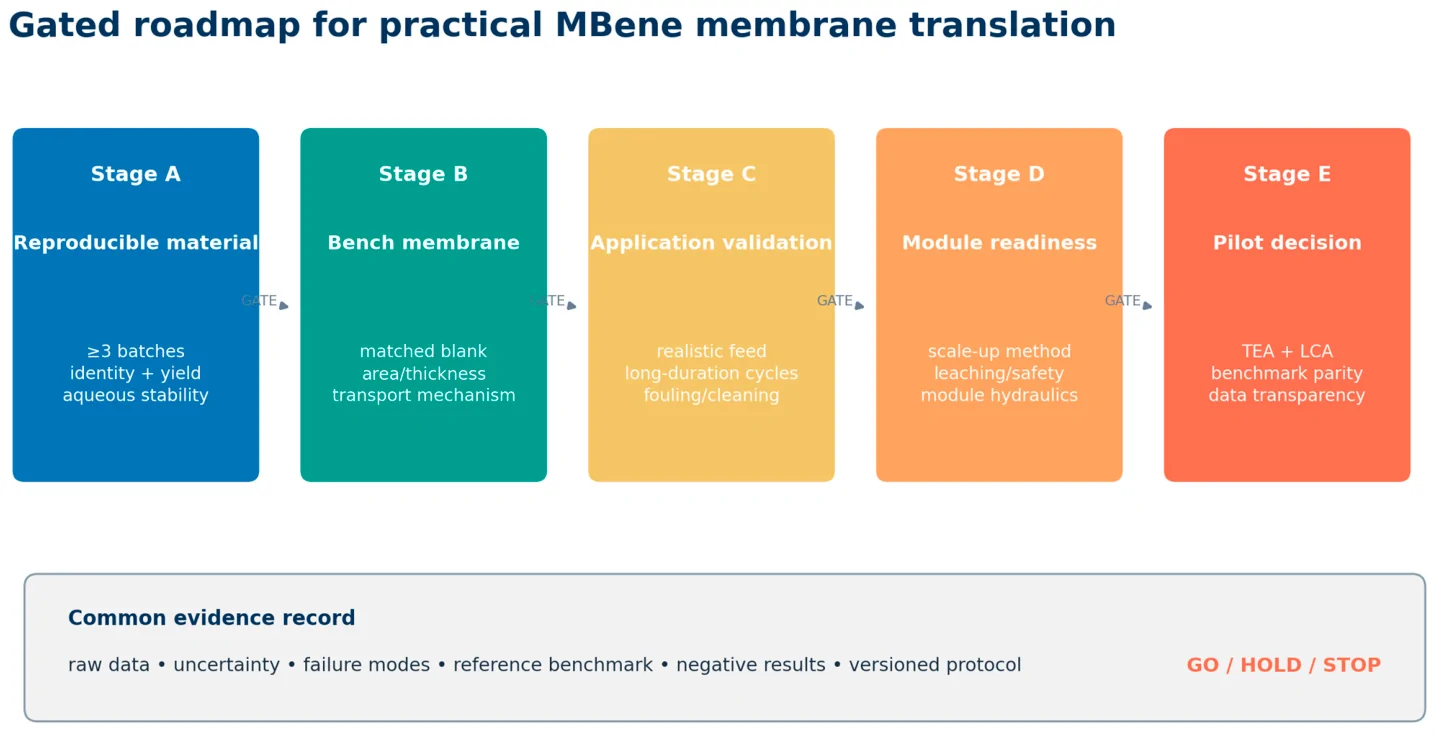
Staged roadmap from reproducible MBene production to a pilot-scale decision. Progress is conditional on passing predefined evidence gates, including durability, safety, benchmark, economic, and life-cycle criteria [109,110,111,112,113,114,115,116,117,118,119,120].

Figure 5 converts the research gap into a gated translation pathway. Stage A establishes a reproducible material rather than a single successful batch. At least three independently prepared batches should be compared for phase identity, composition, nanosheet dimensions, yield, and aqueous aging, with acceptance limits stated before membrane testing. This follows from the route sensitivity demonstrated for Mo-Al-B conversion and exfoliation [109,110,111,112]. Stage B creates a bench membrane and tests causality. A matched blank, identical support and fabrication history, measured active area and thickness, and replicated coupons are needed to isolate the MBene contribution. Stage C evaluates the intended application using a defined synthetic and then representative real feed, extended operation, fouling and cleaning cycles, mass balance, and post-test characterization. Standardized methodology is essential because changes in temperature, concentration, hydrodynamics, pressure, and membrane orientation can prevent valid cross-study comparison [118,120]. Stage D addresses module readiness: scalable deposition, material utilization, support compatibility, pressure tolerance, leaching, worker safety, waste handling, and module hydraulics. Stage E is a decision point rather than an assumption of commercialization. Techno-economic and life-cycle analyses should use measured material, energy, replacement, and waste data and compare the design against an appropriate commercial benchmark. A design that loses selectivity after cycling, releases material, or cannot meet benchmark performance should be redesigned before scale-up. Maintaining raw data, uncertainty, negative results, and versioned protocols prevents selective reporting and allows independent reproduction. Figure 5, therefore, defines progress by completed evidence gates, not by publication count or optimistic language [119,120].

Table 5 provides a minimum reporting set for future MBene membrane studies. Its purpose is to make results interpretable and repeatable, not to prescribe one process. Material identity requires precursor and product phase data, composition, surface chemistry, flake dimensions, yield, and independently synthesized batches because MBene routes can generate different intermediates and products [109,110,111,112]. Membrane construction must state the configuration, support, deposition method, MBene loading, active area, relevant dry or wet thickness, conditioning, and defect-assessment method. A matched membrane without MBene and independently fabricated coupons are necessary to attribute changes to the added material. Feed and operation records should include every solute and concentration, pH, conductivity, temperature, pressure or osmotic gradient, hydrodynamics, recovery, stabilization criterion, and sampling schedule. These variables are essential because membrane coefficients and apparent rejection depend on test boundaries [118,120]. Performance should be reported as time-resolved flux and normalized permeance where appropriate, together with rejection or selectivity definitions, uncertainty, replicates, and a mass balance. Durability extends beyond a short constant-feed test: wet aging, repeated start–stop and cleaning cycles, oxidation, leaching, and before/after structural characterization are needed, as stability-focused MXene work illustrates [116]. Finally, translation claims require measured material and energy inventories, scale-up yield, waste and reagent recovery, module assumptions, a stated techno-economic and life-cycle boundary, and sensitivity analysis against a commercial comparator [119,120]. Unmeasured requirements should be reported as unavailable rather than replaced by estimates from another material. This checklist supports comparison, meta-analysis, and rational go/hold/stop decisions.

## 6. Conclusions

This review combined an author-generated bibliometric analysis with a critical assessment of the prospective use of MBenes in water-treatment and desalination membranes. The 279 Scopus-indexed records retrieved on 14 April 2026 show strong growth in MBene research, particularly in synthesis, computational modeling, catalysis, and energy storage. Direct MBene solar-evaporation studies are now acknowledged, but pressure-driven desalination and filtration evidence remains scarce, leaving a substantial gap between material-level or predicted properties and validated RO, NF, or UF membrane performance [5,6,8,12,13,37].

Available studies indicate composition-dependent electrical, structural, surface, catalytic, and adsorption properties that may be relevant to membrane design [8,9,10,11,12,13,21,46]. Direct evidence for MBene membrane permeability, salt rejection, fouling resistance, antibacterial activity, oxidation stability, and long-term durability is nevertheless insufficient [8,9,10,11,12,13,21,46]. MXene, graphene, GO, and TMD membranes provide useful analogous evidence, but those results do not validate equivalent MBene performance [8,9,10,11,12,13,21,46].

Future research should prioritize controlled synthesis and characterization of true MBenes, reproducible incorporation into polymeric or lamellar membranes, and comparisons with established materials under identical conditions [6,7,9,10,11,12,13,39]. Essential measurements include purity, surface chemistry, dispersion, aggregation, permeability–selectivity relationships, fouling and cleaning behavior, leaching, toxicity, mechanical stability, and long-term operation with realistic feeds [6,7,9,10,11,12,13,39]. Pilot testing, lifecycle assessment, and techno-economic analysis are required before sustainability, cost-effectiveness, or commercial-feasibility claims can be supported [6,7,9,10,11,12,13,39].

Overall, MBenes are promising but experimentally underdeveloped candidates for advanced water-treatment and desalination membranes [6,7,12,13,43]. Their relevance will depend on transparent, standardized, and reproducible studies that connect composition and synthesis to verified membrane performance [6,7,12,13,43].

## Figures and Tables

**Table 1:** Comparative assessment of major two-dimensional materials for desalination membrane applications.

Material	Membrane Configuration	Process, Feed, and Test Conditions	Reported Performance	Evidence Level	Refs.
Graphene	Oxygen plasma–etched, suspended nanoporous single-layer graphene	Pressure-driven water transport at 40 °C and an estimated pressure difference of approximately 17 kPa; separate osmotic-pressure experiments	Nearly 100% salt rejection for low-defect-density membranes; water flux up to approximately (10^6^) g m^−2^ s^−1^ under the pressure-driven experimental configuration	Direct experimental evidence; specialized nanoscale configuration	[10,56]
Graphene oxide–graphene	Approximately 5-micrometer-thick swelling-controlled GO–graphene laminate	Forward osmosis; 0.1 M NaCl feed and 3 M sugar draw solution	Approximately 97% NaCl rejection and 0.5 L m^−2^ h^−1^ water flux	Direct experimental evidence	[57]
MoS_2_	Approximately 1-micrometer-thick peptide-functionalized porous MoS_2_ nanosheet/nanodisk laminate on an alumina support	Forward osmosis; 0.5 M NaCl feed and 2 M sucrose draw solution; continuous testing	>99% initial NaCl rejection; >95% rejection over 30 days; approximately 5 L m^−2^ h^−1^ water permeance for the selected laminate	Direct experimental evidence	[11,60]
Ti_3_C_2_T_x_ MXene	Approximately 1.1-micrometer-thick Al^3+^-intercalated Ti_3_C_2_T_x_ lamellar membrane supported on PES	NaCl solutions from 0.2 to 2.0 M and synthetic seawater containing KCl, NaCl, Na_2_SO_4_, CaCl_2_, and MgCl_2_; aqueous testing up to 400 h	Approximately 89.5–99.6% NaCl rejection and 1.1–8.5 L m^−2^ h^−1^ water flux, depending on membrane and feed conditions	Direct experimental MXene evidence; not MBene evidence	[9,22]
MBenes	MoAl_1−x_B photothermal layer integrated with a thermally insulated nylon support	Solar interfacial steam generation under one-sun irradiation; seawater, brine, and selected contaminated-water feeds	Evaporation rate of 1.59 kg m^−2^ h^−1^ and reported efficiency of 96.66% under one-sun irradiation	Direct experimental solar-evaporation evidence; not a pressure-driven RO, NF, or UF test	[12]

**Table 2:** Evidence-based comparison of major two-dimensional material classes investigated for membrane-based water treatment.

Material Class	Membrane-Level Evidence	Experimentally Demonstrated Findings	Principal Limitations of Interpretation	Evidence Classification	Refs.
Graphene	Available for nanoporous single-layer membranes	High salt exclusion and rapid water transport have been demonstrated in specialized nanoscale experimental systems	Small active area, defect control, unusual test geometry, and scale-up limitations prevent direct comparison with conventional membranes	Direct experimental membrane evidence	[10,56]
Graphene oxide	Available for lamellar and composite membranes	Controlled interlayer spacing can enable ion sieving and high NaCl rejection	Performance depends strongly on swelling control, membrane thickness, cross-linking, feed concentration, and operating mode	Direct experimental membrane evidence	[52,53,57,59]
TMDs, particularly MoS_2_	Available for functionalized and porous lamellar membranes	Salt rejection, water transport, and extended aqueous stability have been experimentally demonstrated	Performance varies with functionalization, surface charge, pore size, interlayer structure, support, and FO or RO operation	Direct experimental membrane evidence	[11,54,60,62]
MXenes, particularly Ti_3_C_2_T_x_	Available for lamellar, intercalated, and composite membranes	Ion sieving, water transport, antibacterial activity, and swelling control have been experimentally investigated	Oxidation, swelling, termination chemistry, intercalation, membrane thickness, and long-term stability remain condition-dependent	Direct experimental MXene membrane evidence	[9,21,22,107]
MBenes	Available for solar interfacial evaporators; insufficient for pressure-driven desalination membranes	Photothermal evaporation and freshwater production have been demonstrated in layered and composite MBene systems	Solar evaporation is not directly comparable with RO, NF, or UF; composition, support, irradiance, feed, and collection conditions differ	Direct experimental solar-evaporation evidence; pressure-driven membrane performance not established	[12,13,43]

**Table 3:** Evidence matrix separating direct MBene observations from computational, analogous, and translational evidence. Absence of pressure-driven MBene membrane data is reported explicitly rather than inferred [109,110,111,112,113,114,115,116,117,118,119,120,121].

Evidence Level	What Is Currently Available	Permitted Interpretation	Evidence Still Required	Refs.
Direct MBene water application	Early MBene solar-evaporation studies are application-specific.	Demonstrates photothermal water production in the tested architecture.	Independent replication; water quality, salt management, durability, and standardized energy balance.	[121]
Direct MBene membrane	No validated pressure-driven MBene RO/NF/UF dataset was identified.	Report as an explicit evidence gap; do not estimate performance from analogous evidence.	Defined membrane composition, active area, feed, pressure, temperature, duration, and matched control.	[118,120]
MBene material experiments	Multiple precursor-conversion and exfoliation routes with structural characterization.	Supports claims about synthesis feasibility and route-dependent composition.	Batch yield, impurity balance, surface terminations, aqueous aging, and reproducibility.	[109,110,111,112]
Computational MBene evidence	Predicted structures and application-relevant properties.	Generates mechanisms and screening priorities.	Experimentally verified structure, defects, terminations, and transport under realistic water chemistry.	[117,119]
2D-material analogous evidence	MXene and graphene studies demonstrate tunable nanoscale transport.	Guides variables, controls, and characterization choices.	Direct testing with true MBenes under matched conditions.	[113,114,115,116,117]
Translation evidence	No MBene membrane pilot or module-level evidence.	Defines a research target, not a present capability.	Scale-up, long-term operation, safety, TEA and LCA against commercial benchmarks.	[119,120]

**Table 4:** Synthesis-to-testing matrix linking MBene preparation variables to material verification, membrane integration, and performance evaluation [109,110,111,112,113,114,115,116,117,118,119,120].

Stage/Route	Variables to Record	Required Verification	Membrane Relevance	Refs.
Topochemical conversion	Parent phase; reagent sequence; temperature; time; separation yield.	XRD phase analysis; microscopy; composition; mass balance.	Residual precursor or oxide can change transport and apparent stability.	[109]
Controlled decomposition	Atmosphere; heating profile; precursor size; product porosity.	Phase fractions; pore-size evidence; surface area; morphology.	Porosity may aid transport but must be distinguished from interflake defects.	[110]
Gaseous-HCl conversion	Gas concentration/flow; temperature; exposure; washing.	Conversion extent; residual Al/Cl; particle dimensions; yield.	Scalability and safe reagent handling must be assessed before membrane fabrication.	[111]
Alkaline hydrothermal etching	Base concentration; solid/liquid ratio; time; temperature; delamination.	XRD, XPS, AFM/TEM; surface chemistry; colloidal stability.	Termination and flake-size distributions affect dispersion and channel assembly.	[112]
Membrane assembly	Support; deposition method; loading; thickness; drying; cross-linking.	Cross-section; defects; wet spacing; adhesion; active area.	Matched blanks isolate the MBene contribution from support and processing effects.	[113,114,115,116]
Transport testing	Feed composition; pressure/osmotic gradient; temperature; flow; duration.	Permeance/flux; rejection/selectivity; uncertainty; mass balance; repeats.	Common conditions and definitions are necessary for defensible comparisons.	[118,120]

**Table 5:** Minimum reporting and validation requirements for MBene membrane studies. The checklist separates material identity, membrane construction, operating conditions, performance, durability, safety, and translation [109,110,111,112,113,114,115,116,117,118,119,120].

Reporting Domain	Minimum Information	Required Controls/Replication	Decision Output	Refs.
Material identity	Precursor and product phases; composition; terminations; flake size/thickness; yield.	≥3 independent batches; retained raw patterns/spectra; impurity and mass balance.	Reproducible MBene identity or return to synthesis.	[109,110,111,112]
Membrane construction	Configuration; support; deposition; loading; active area; dry/wet thickness; conditioning.	Matched support/polymer blank; replicated coupons; defect inspection.	Attributable membrane structure or redesign.	[113,114,115,116]
Feed and operation	Solutes; concentrations; pH; conductivity; temperature; pressure/gradient; flow; recovery.	Calibrated instruments; stabilization criterion; complete mass balance.	Comparable test boundary conditions.	[118,120]
Performance	Flux and permeance; rejection/selectivity; uncertainty; time-resolved data.	≥3 independent membranes; commercial or established benchmark under the same conditions.	Effect size with confidence and benchmark position.	[118,119,120]
Durability and safety	Continuous duration; cycles; cleaning; wet aging; oxidation; leaching; post-test structure.	Blank leaching control; before/after chemistry; failure-mode record.	Stable, recoverable, and contained performance or hold.	[116,120]
Translation	Material/energy inventory; scale-up yield; waste; module assumptions; TEA/LCA boundaries.	Sensitivity analysis; transparent baseline; commercial comparator.	Go, hold, or stop decision with limiting variables.	[119,120]

## Data Availability

No new data were created or analyzed in this study. Data sharing is not applicable to this article.
